# Photocatalytic Plates for Production of Hydrogen and Value-Added Products via Glycerol Photoreforming

**DOI:** 10.1021/acsomega.5c09097

**Published:** 2026-02-03

**Authors:** Mayara M. R. Oliveira, Emanoel J. R. Sousa, Luana S. Bomfim, Mariana M. Duarte, Antonio J. M. Sales, Renato A. Antunes, Sydney F. Santos, F. Murilo T. Luna, Rinaldo S. Araújo, Peter K. J. Robertson, Bruno C. B. Salgado

**Affiliations:** 1 Federal University of Ceará, Av. Mister Hull, s/n, Pici, Fortaleza, Ceará 60.455-760, Brazil; 2 Federal Institute of Education, Science and Technology of Ceará, Av. Parque Central, 1315, Distrito Industrial, Maracanaú, Ceará 61.939-140, Brazil; 3 Federal University of ABC, Av. dos Estados, 5001, Bangu, Santo André, São Paulo 09.280-560, Brazil; 4 Queen’s University Belfast, Stranmillis Road, Belfast BT9 5AG, United Kingdom

## Abstract

Photocatalytic hydrogen production has emerged as a promising strategy due to the potential of using renewable sources such as sunlight and biomass. The scalability of this process depended on the optimization of the reaction system design, as well as on the reduction of costs and time required for the catalyst separation, purification, and reuse steps. This study presents the application of photocatalytic plates with immobilized TiO_2_ doped with low amounts of platinum (Pt) at the photoreforming of glycerol under visible-light irradiation. Catalysts were synthesized via photodeposition and characterized using SEM, XRD, BET, and impedance spectroscopy. The results demonstrated that the photodeposition method promoted the formation of nanometric Pt particles on the TiO_2_ surface, significantly increasing H_2_ production and charge separation efficiency compared to results obtained using pure TiO_2_. This improvement was evidenced by the increase in the H_2_ evolution rate and the formation of high-value products, such as glyceraldehyde and dihydroxyacetone. The reaction temperature proved to be an essential factor for optimizing the reaction rate. The photocatalytic activity, however, already reached satisfactory performance at 40 °C, eliminating the need for additional heating to increase hydrogen production. The main byproducts identified reinforce the versatility of photocatalysis as an efficient route for sustainable glycerol valorization.

## Graphical abstract

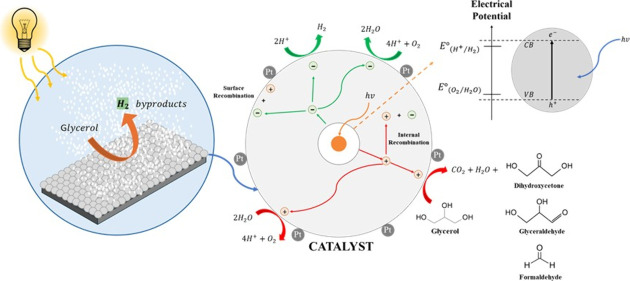

## Introduction

The growing scarcity of fossil fuels and rising CO_2_ emissions have driven the search for renewable energy sources and more sustainable processes.^1^ In this context, hydrogen (H_2_) stands out as a promising alternative, having wide industrial applications, being able to replace fossil fuels in heat generation, steelmaking, the production of fertilizers, and the manufacture of materials such as ceramics and glass and chemical inputs.^2,3^ Furthermore, it can act as an energy vector, providing electricity and enabling fuel cell mobility systems.

Hydrogen production involves different raw materials, routes, and technologies, including sources derived from fossil fuels, such as oil, natural gas, and coal, and renewable sources, such as water electrolysis. Global H_2_ production in 2023 reached 97 Mt, an increase of 2.5% compared to 2022. Hydrogen demand remains concentrated in traditional applications, namely, refining, the chemical sector (ammonia and methanol production), and steel manufacturing (to produce iron via the direct reduced iron (DRI) route using fossil-based synthesis gas). This demand is almost wholly met with hydrogen produced from unabated fossil fuels.^4^

In this way, the cost of producing H_2_ and the concept of clean energy go opposite since using raw materials, as coal and oil, releases a significant amount of greenhouse gases into the atmosphere. CO_2_ emissions associated with hydrogen production and use increased to 920 Mt CO_2_, 1.5% greater than in 2022, and equivalent to the annual emissions of Indonesia and France combined. Considering this, heterogeneous photocatalysis emerges as a promising technology, as it enables the utilization of renewable and abundant resources to produce H_2_ more sustainably.^5–8^

The photocatalytic process for hydrogen generation occurs through the acquisition of photons by a photoactive material, typically a semiconductor, promoting the movement of electrons, which migrate from the valence band (VB) to the conduction band (CB). These excited electrons participate in the reduction of hydrogen ions. Simultaneously, the photogenerated holes left in the VB act as oxidation sites for water and a sacrificial agent, such as glycerol. Glycerol acts as an electron donor, suppressing electron–hole recombination and thereby enhancing the generation of H_2_.^6,8–11^

The production of H_2_ from photoreforming of glycerol is advantageous due to its high stoichiometric yield, low cost, and good solubility in water.^12,13^ Santoso et al.^14^ evaluated the influence of glycerol concentration on the photocatalytic production of H_2_. The results showed that a concentration of 10% (v/v) increased hydrogen production up to by 6-fold compared to the reaction conducted with water alone. Eisapour et al.^15^ showed the formation of high-value-added products, such as glyceraldehyde, dihydroxyacetone, and formaldehyde, parallel to the generation of H_2_ from glycerol photoreforming. These byproducts are economically more interesting than glycerol itself. Glyceraldehyde can be applied in the cosmetics, pharmaceutical, and organic chemical industries.^16^ Dihydroxyacetone is used in the cosmetics industry and as a monomer in the manufacture of biomaterials in the polymer industry.^17^

Titanium dioxide (TiO_2_) is the main photocatalyst used due to its high photoactivity, low cost, low toxicity, and excellent stability. For these reasons, it has been widely applied in processes such as air purification and pollutant degradation.^18^ Its efficiency under sunlight is, however, limited due to its wide bandgap (3.2 eV, 380 nm), restricting its application to the UV part of the spectrum. Furthermore, it has a fast charge recombination rate, reducing the overall efficiency of the process. To address these limitations, it is essential to adjust its structure using suitable methods.^19^ Platinum (Pt) doping is one highly effective method. The presence of Pt as a dopant tends to facilitate electron transport and significantly reduces electron/hole pair recombination.^20–22^

It is important to highlight that studies on photocatalytic hydrogen production are generally carried out in discontinuous occurrence systems, where the photocatalyst is applied as a suspension, aiming to guarantee its uniform distribution. The subsequent steps, such as separation, purification, and reuse of the catalyst, would make it difficult to scale up the process. Several studies have addressed these challenges, with a primary focus on hydrogen production from wastewater and feedstock waste. Valencia-Valero et al.^23^ developed an outdoor prepilot-scale flat solar panel photocatalytic reactor. The system comprised an effective irradiated area of approximately 0.14 m^2^ and was operated outdoors under natural sunlight in a continuous circulation mode. Performance evaluation was carried out using glycerol as a sacrificial reagent and a real wastewater effluent. In another study of the same group,^24^ the application of solar photoreforming to food industry effluents, aiming to produce hydrogen and simultaneously degrade oxygenated organic compounds. This study employed the same flat solar panel photocatalytic reactor, with emphasis placed on improving the performance of TiO_2_-based photocatalysts through adjustments in the synthesis methodology and careful selection of cocatalysts. Based on the same principle, other studies have used immobilization techniques, such as Bhattacharjee et al.,^25^ who proposed a chemoenzymatic photoreforming approach integrating enzymatic pretreatment of polyester plastics with photocatalytic hydrogen production under mild operating conditions. A thin TiO_2_–Pt photocatalyst film was deposited on glass substrates by drop-casting, resulting in reusable catalytic sheets integrated into a custom-built, sealed reactor equipped with a quartz window. The study carried out by Uekert et al.^26^ also used the same immobilization technique. However, the focus of the work was to examine scalable solar photoreforming systems utilizing immobilized photocatalyst panels for hydrogen production from plastic, biomass, and mixed waste streams. Additionally, Nishiyama et al.^27^ reported a large-scale demonstration of photocatalytic solar hydrogen production from water, utilizing a 100 m^2^ outdoor flat-panel reactor system under realistic field conditions. The Al/SrTiO_3_ photocatalyst was immobilized as a thin particulate sheet on glass substrates by spray coating. The system operated under natural sunlight, without a sacrificial reagent.

As previously discussed, glycerol can be catalytically converted into a variety of high-value-added products, and, as the main waste stream of the biodiesel industry, it also stands out as an attractive feedstock to photocatalytic hydrogen production. Our study proposes a simple method for the preparation of immobilized TiO_2_ catalyst doped with Pt, designed for the photoreforming of glycerol. We also evaluate the influence of the temperature on catalyst activity. The results demonstrate that the immobilized catalyst emerges as a promising material for biomass valorization.

## Materials and Methods

### Reactants

Titanium dioxide (TiO_2_) supplied by Evonik (Degussa, P25—80% anatase and 20% rutile) was used as a catalyst. Pt doping was carried out from its precursor: hexachloroplatinic acid (IV) hexahydrate (H_2_PtCl_6_·6H_2_O, Sigma-Aldrich); Nafion 117 (5%) was used as an adhesive agent to immobilize the material on the plates. Dinâmica supplied glycerol.

### Platinum Deposition on Titanium Dioxide (TiO_2_)

For the study, photocatalysts made of titanium dioxide modified with platinum (0.3%, w/w) were synthesized following the procedure described by Oliveira et al.^28^ Thus, a mass of hexachloroplatinic acid (IV) hexahydrate (H_2_PtCl_6_·6H_2_O) was dissolved in an aqueous solution of methanol (10% v/v) in the presence of TiO_2_ P25, according to the metal content relative to TiO_2_ (m/m). The suspension was kept under stirring and irradiated with a 300 W xenon lamp for 3 h. After the photodeposition, the as-prepared material was centrifuged and washed with distilled water until complete removal of chloride. The recovered material was finally dried at 80 °C. The material obtained was called TiO_2_@Pt_0.3_.

### Immobilization on Photocatalytic Plates

The photocatalytic plates were made of acrylic polymer materials (5.0 × 1.0 cm). To immobilize the catalyst, a solution was prepared by mixing 25 mg of the catalyst with 25 μL of a solution containing Nafion 117 (5%) and 500 μL of ethyl alcohol and homogenized via a probe sonicator. Subsequently, the solution was gently spread on the acrylic plate and heated at 40 °C for 1 h to evaporate the solvent.

### Characterization of Photocatalysts

The characterization of the synthesized materials was carried out using N_2_ adsorption/desorption experiments at 77 K, using Autosorb iQ3 equipment (Quantachrome Instruments). The samples were previously degassed under reduced pressure at 200 °C for 2 h. The BET (Brunauer–Emmet–Teller) and BJH (Barret–Joyner–Halenda) models were adopted for data interpretation. XRD analyses were performed on a PANalytical XPert Pro MPD diffractometer. Measurements were obtained in an angular range of 10–90° (2θ) using a Co Kα radiant source (40 kV and 45 mA). The diffuse reflectance spectrum of the synthesized materials was obtained using Thermo Evolution 300 equipment, performing a spectral scan from 300 to 800 nm. The dielectric properties of the material were obtained by impedance spectroscopy in the radiofrequency range. For impedance spectroscopy analysis, the samples were sintered in cylindrical molds approximately 15 mm in diameter, where they were subjected to a pressure of 110 MPa in a hydraulic press, acquiring a thickness of approximately 1.5 mm. Before pressing the samples, approximately 5% by mass of PVA binder (poly­(vinyl alcohol), 10% v/v) was added to the ceramic powder to promote plasticity and reduce brittleness during removal from the mold. The sintering heat treatment was carried out at 480 °C for 2 h at a rate of 2 °C/min. The sintering temperature was chosen to avoid reaching the crystalline phase transition temperature limit of titanium oxide (550 °C), where the transition from the anatase crystalline phase to the rutile crystalline phase would occur. An impedance analyzer (model Solartron 1260) was used as a function of frequency (1 Hz–10 MHz) and temperature (400 °C). X-ray photoelectron spectrometry measurements were conducted by Specs XPS/USP system with a Phoibos 150 analyzer and CMOS 2D detector with an Al Kα radiation source. High-resolution transmission electron microscopy (HR-TEM), scanning transmission electron microscopy (STEM) with a high-angle annular dark-field (HAADF) detector, and energy-dispersive X-ray spectroscopy (EDX) maps were acquired in a Thermo Scientific microscope, model Talos F200X G2 scanning transmission electron microscope ((S)­TEM) operating at 200 kV and equipped with an EDX spectrometer (four detectors) model Super-X EDS.

### Photocatalytic Reactions for H_2_ Production

The photocatalytic tests were carried out in a multiple simultaneous reaction system (MSR) (BR 1020210170980), which allows the execution of up to eight tests under different operational conditions, using borosilicate glass flasks with silicone septa to collect the gas phase (detailed schematic in Figure S1). At the center of the reactor, a xenon lamp (300 W) positioned equidistant from the flasks provided the irradiation. The irradiance of the xenon lamp was measured at the catalyst position using a Metrohm Autolab PGSTAT204 potentiostat coupled to the optical bench, yielding a value of 109.4 mW·cm^–2^, corresponding to standard one-sun conditions (100 mW·cm^–2^). 25 mL of 10% (w/v) glycerol solution was transferred to the reaction flasks with the photocatalytic plates, previously purged with N_2_ to eliminate O_2_ and ensure an inert atmosphere. The reactions were carried out for 3 h, with an integrated cooling system to maintain the temperature at approximately 40 °C. Figure 1 shows the emission spectrum of the Xe lamp obtained with the CCS200 spectrometer (Thorlabs).

**Figure 1:**
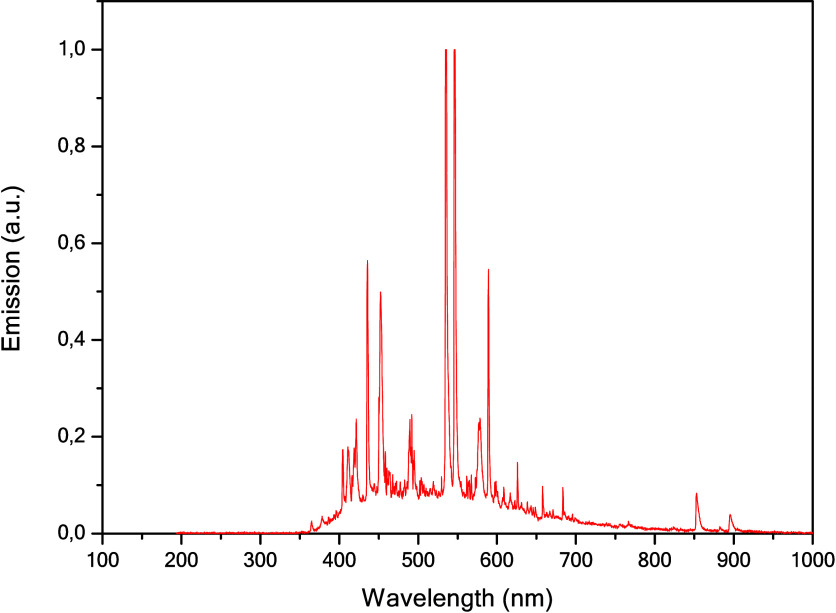
Spectrum of the xenon lamp (300 W) used in photocatalytic reactions.

The quantitative determination of H_2_ was conducted employing a micro-GC 490 (Agilent Technology) equipped with a TCD detector. The equipment has a Pora PlotU column (10 m) and uses nitrogen (N_2_) as a carrier gas.

The kinetic study of H_2_ production was carried out in a 250 mL Kitasato flask at 20, 40, and 60 °C, with temperature control based on the liquid phase of the reaction medium. An aqueous glycerol solution (10%, v/v) was placed in contact with a plate containing 25 mg of immobilized catalyst. Prior to the reaction, the system was purged with N_2_ to remove dissolved O_2_ and establish an inert atmosphere. The photocatalytic reaction was conducted under irradiation from a 300 W xenon (Xe) lamp, and the produced hydrogen was collected at 15 min intervals for kinetic monitoring.

### Byproduct Analysis

Liquid-phase byproducts were identified and quantified by liquid chromatography (Shimadzu LC-2050C 3D), equipped with a Rezex ROA column (8%). A solution of H_2_SO_4_ (0.5 mM) and acetonitrile (70:30) was used as a mobile phase under a flow rate of 0.4 mL·min^–1^ and an oven temperature of 40 °C. Aliquots of 30 μL of the liquid fraction were analyzed to identify and quantify the components at 190 nm.

## Results and Discussion

### Characterization of Photocatalysts

Table 1 describes the textural characteristics of the catalysts evaluated (TiO_2_ and TiO_2_@Pt_0.3_) using the BET and BJH methods. According to Figure 2, both catalysts presented type IV isotherms, with H3 hysteresis in a relative pressure range of 0.4 to 1.0, typical of materials with a mesoporous structure.^29,30^ The specific surface area was slightly reduced with Pt doping before increasing again.

**Figure 2:**
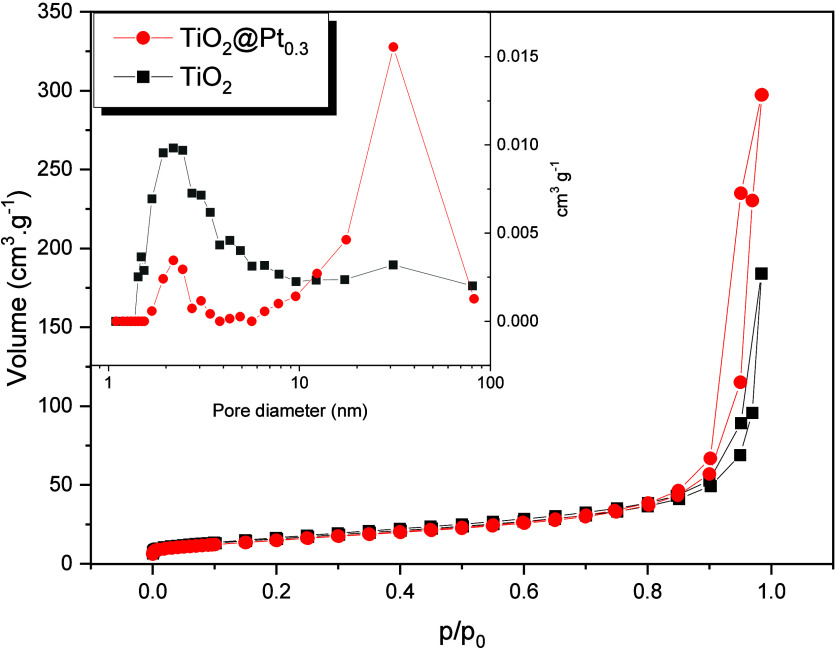
N_2_ adsorption/desorption isotherms and pore distribution curves (inset) of TiO_2_ and TiO_2_@Pt_0.3_.

**Table 1:** Textural Characteristics of TiO_2_ and TiO_2_@Pt_0.3_

catalyst	surface area (m^2^ g^–1^)	pore diameter (nm)	pore volume (cm^3^ g^–1^)
TiO_2_	64.96	2.19	0.286
TiO_2_@Pt_0.3_	63.45	31.06	0.465

The rapid increase in pore diameter suggests that Pt was preferentially deposited in existing TiO_2_ pores and subsequently formed new ones on the surface through overlapping layers, as suggested by the change in pore volume. This would lead to an increase in the adsorption capacity of the catalyst, subsequently improving the efficiency of the photocatalytic process.^31^ Thus, the characterization suggests that Pt doping would not only improve catalytic activity, by generating additional active sites, but also induce modifications on some of the textural properties of the catalyst.

The diffractogram profiles shown in Figure 3a indicate that the materials have well-defined crystallinity, with narrow peaks indicating large crystallite sizes. Pt deposition at the employed concentration did not lead to significant changes in the crystallinity of TiO_2_, and no peak was detected regarding Pt. The samples were found to contain anatase and rutile in an 80:20 ratio, with the most prominent reflections appearing at 2θ of 29° (101) and 32° (110), confirmed by their respective reference standards. There were shifts in the diffraction peak shift of TiO_2_, indicating that the metals were located in the (101) plane of TiO_2_ (Figure 3b).

**Figure 3:**
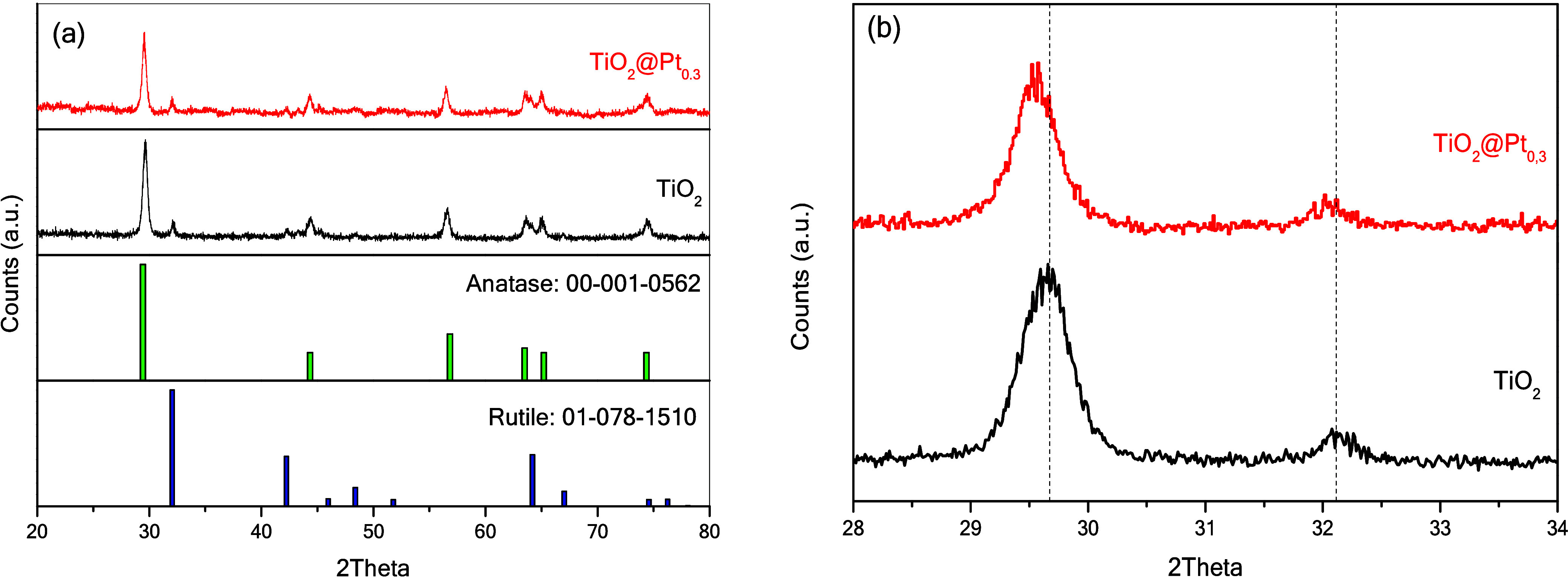
(a) XRD profiles of TiO_2_ and TiO_2_@Pt_0.3_ and (b) band shift due to platinum coating.

HR-TEM images of the TiO_2_@Pt_0.3_ sample are shown in Figure 4a,b. In Figure 4a, it is possible to observe a representative region of the investigated sample. TiO_2_ nanoparticles with sizes ranging from around 20 to 50 nm can be observed. Small-rounded particles of Pt can also be observed. Figure 4b shows a Pt nanoparticle deposited on the surface of a TiO_2_ particles with size close to 10 nm.

**Figure 4:**
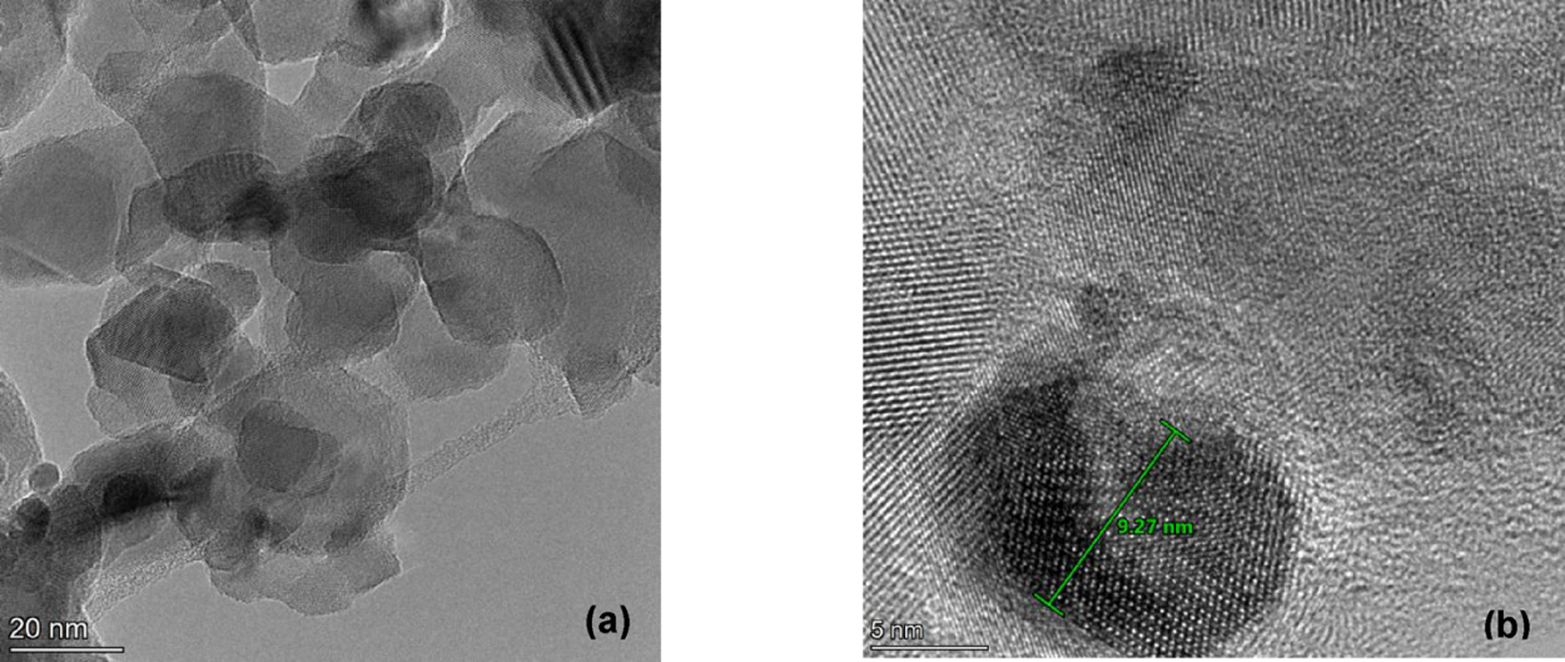
HR-TEM images of the TiO_2_@Pt_0.3_ sample. (a) General view of the microstructure and (b) Pt nanoparticle.

Figure 5 shows HAADF-STEM images and an EDX map for the TiO_2_@Pt_0.3_ sample. The atomic number (*Z*) contrast observed in the HAADF detector indicates that brighter small round nanoparticles are composed of an element with higher Rutherford scattering, in this case, Pt. It is possible to observe a homogeneous dispersion of these nanoparticles throughout larger nanoparticles with smaller Rutherford scattering, which can be attributed to TiO_2_. The size of Pt nanoparticles ranges from about 5 to 10 nm. To confirm the HAADF-STEM results, the region indicated in the red square was used for EDX mapping. This analysis clearly showed that the small-rounded nanoparticles are indeed Pt dispersed on TiO_2_.

**Figure 5:**
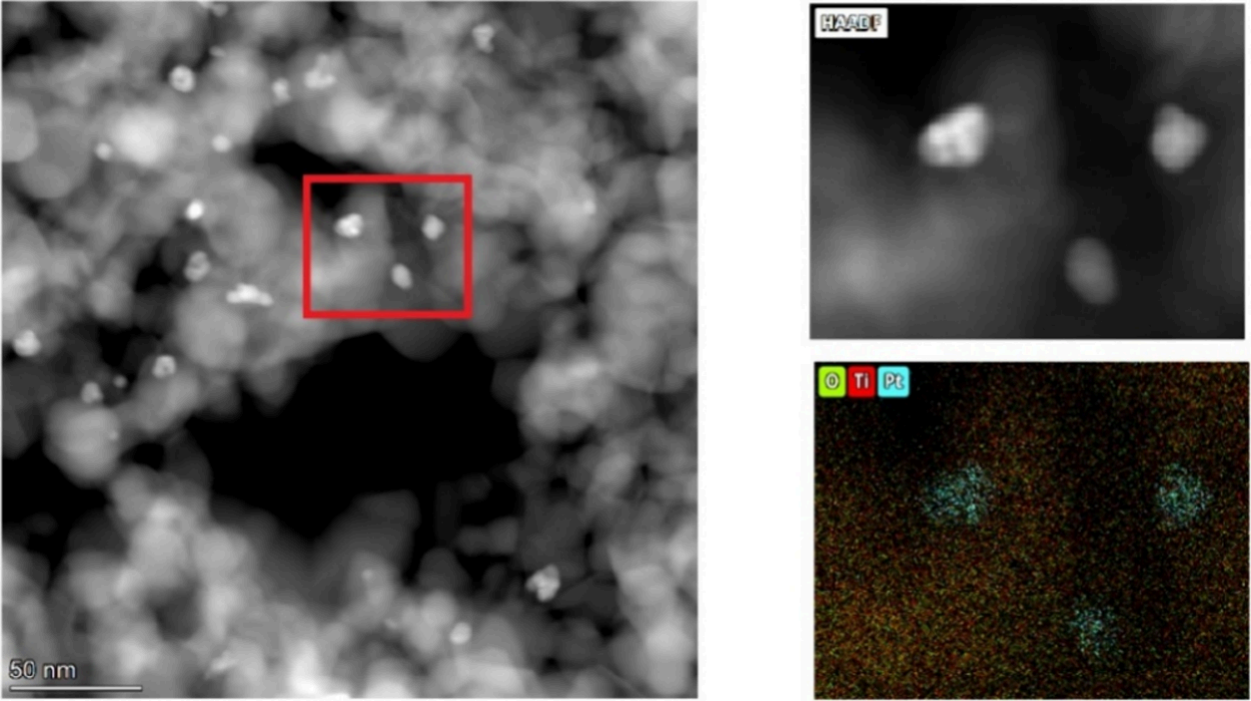
HAADF-STEM image showing the dispersion of Pt nanoparticles throughout the larger TiO_2_ nanoparticles.

The optical properties of the photocatalysts were determined by diffuse reflectance spectroscopy, as shown in Figure 6. The band gap value of the catalysts was determined by the Kubelka–Munk (*F*(*R*)) function, according to their spectral data. Knowing that the TiO_2_ phase has an indirect bandgap,^32^ its value is obtained from the intercept of the graph generated by [*F*(*R*)·*h*ν]^1/2^ versus *h*ν. The band gap values of TiO_2_ and TiO_2_@Pt_0.3_ were 3.32 and 3.10 eV, respectively, confirming the spectral shift to the formation of a Schottky barrier at the metal–semiconductor interface between Pt nanoparticles and TiO_2_.

**Figure 6:**
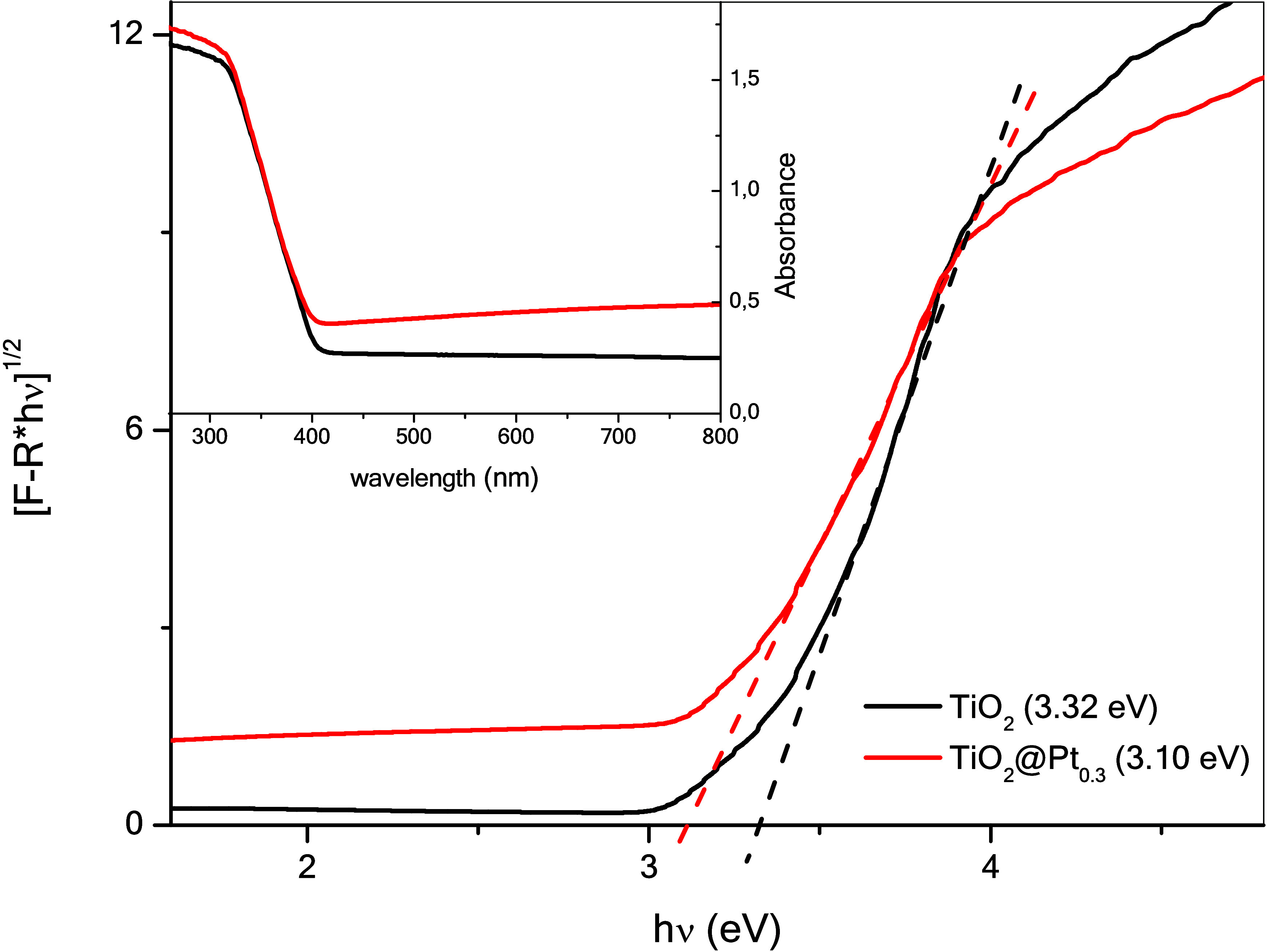
DRS and bandgap of TiO_2_ and TiO_2_@Pt_0.3_.

The potentials of the valence (VB) and conduction (CB) bands were calculated through the Mulliken electronegativity and bandgap of the semiconductor,^33^ according to eqs 1 and 2.

$$E_{\mathrm{VB}}=X-E_{e}+0.5E_{g}$$ 1

$$E_{\mathrm{CB}}=E_{\mathrm{VB}}-E_{g}$$ 2

where *E*_g_, *E*_VB_, and *E*_CB_ are the band gap and potentials of the valence and conduction bands of the semiconductor, respectively, *E*_e_ is the energy of free electrons of the hydrogen scale (4.5 eV), and *X* is the absolute electronegativity (Mulliken) of the atom semiconductor, expressed as the geometric mean of the absolute electronegativity of the constituent atoms, which is defined as the arithmetic mean of the atomic electro affinity and the first ionization energy.

The calculated valence band (*E*_VB_) and conduction band (*E*_CB_) potentials for pure TiO_2_ were 2.97 and −0.35 eV, respectively. For TiO_2_@Pt_0.3_, the apparent shifts to 2.86 eV (*E*_VB_) and −0.24 eV (*E*_CB_) should be interpreted as modifications in the effective band alignment due to Schottky barrier formation, not as fundamental changes in the TiO_2_ electronic structure. At the Pt-TiO_2_ interface, a Schottky barrier forms due to the difference in work functions between metallic Pt (∼5.65 eV) and TiO_2_ (∼4.2 eV for anatase). This creates an internal electric field that promotes efficient electron transfer from the TiO_2_ conduction band to Pt nanoparticles, while the holes remain in the TiO_2_ valence band (Figure 7).

**Figure 7:**
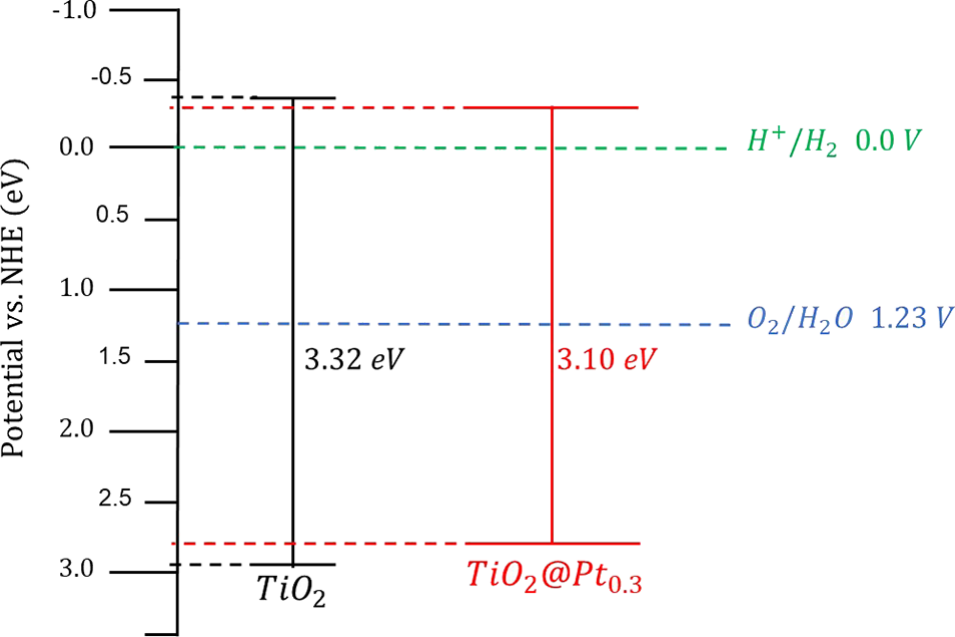
Shifting of the band gap potentials.

Additional characterizations were performed to assess structural and chemical modifications in the spent catalysts. X-ray photoelectron spectroscopy (XPS) was employed to investigate the oxidation states and surface distribution of platinum supported on TiO_2_.

Figure 8 shows the Ti 2p high-resolution spectra for the TiO_2_@Pt_0.3%_ samples before and after reaction. The spectrum obtained for the sample before reaction (Figure 8a) exhibited one single doublet whose peaks were centered at 458.5 and 464.2 eV. The splitting between those peaks was 5.7 eV, typical of the Ti^4+^ chemical state in TiO_2_.^34,35^ After reaction, the Ti 2p spectrum was fitted with two doublets. The first one at lower binding energies was centered at 457.1 and 462.8 eV, being assigned to the Ti^3+^ chemical state, in accordance with the literature.^36^ The second doublet at higher binding energies was also due to Ti^4+^ in TiO_2_.

**Figure 8:**
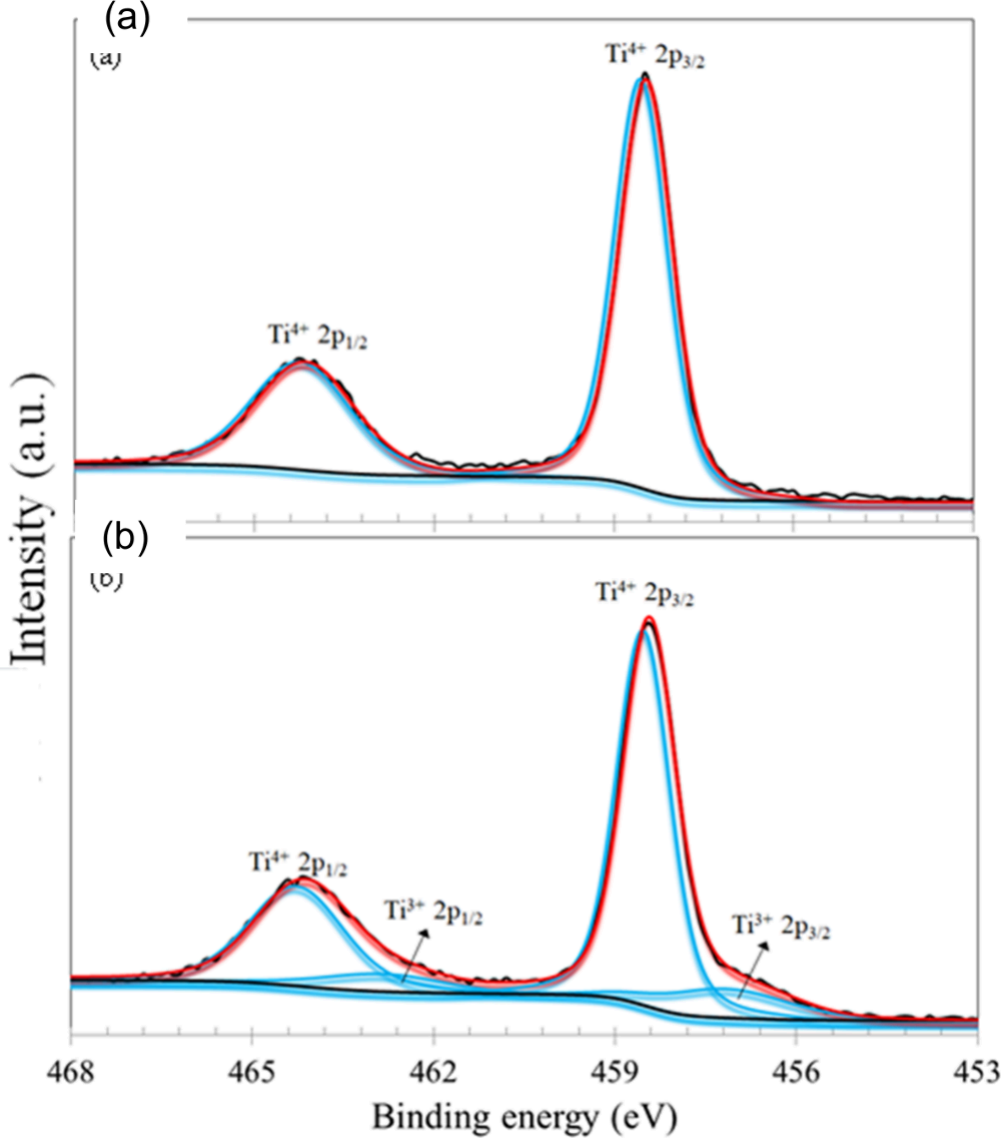
XPS high-resolution spectra of Ti 2p photoelectron lines for the TiO_2_@Pt_0.3%_ samples: (a) before reaction; (b) after reaction.

The O 1s high-resolution spectra for the TiO_2_@Pt_0.3%_ samples before and after reaction are shown in Figure 9. Before reaction (Figure 9a), the spectrum was fitted with three components, assigned to lattice oxygen in the TiO_2_ structure (529.7 eV), adsorbed surface oxygen or oxygen vacancies (531.4 eV), and a small fraction of adsorbed water (532.8 eV), as observed by other authors.^37,38^ The same components were observed after reaction.

**Figure 9:**
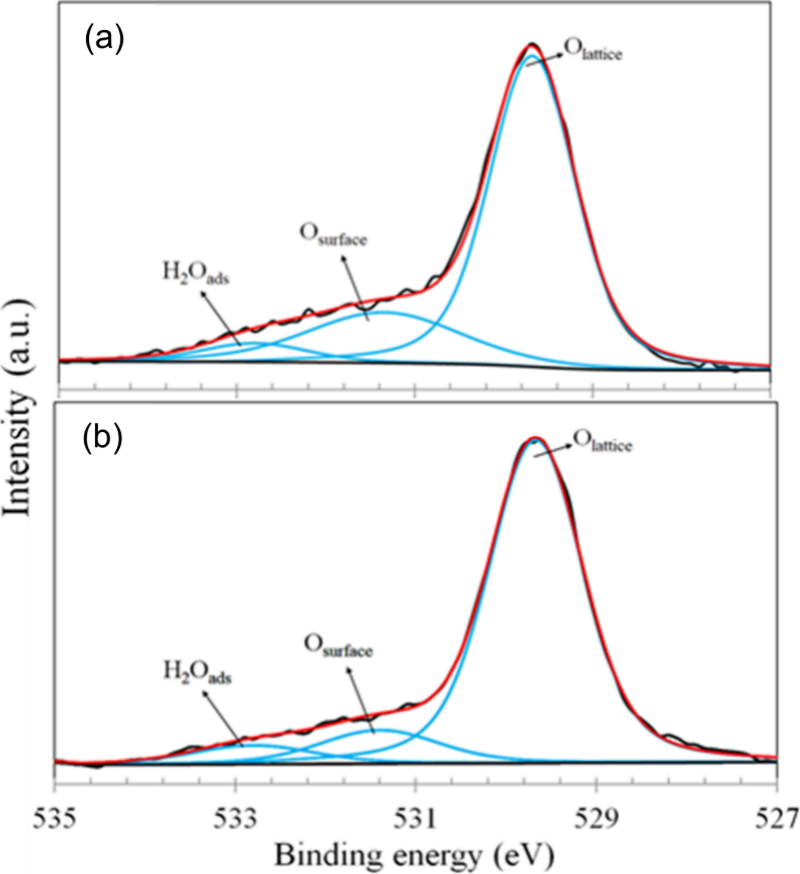
XPS high-resolution spectra of O 1s photoelectron lines for the TiO_2_@Pt_0.3%_ samples: (a) before reaction; (b) after reaction.

The Pt 4f high-resolution spectra are shown in Figure 10. The spectra of both samples are characterized by a low-intensity doublet with asymmetric peak shape, typical of metallic platinum (Pt^0^),^39^ with the spin–orbit 4f_7/2_ and 4f_5/2_ components centered at 70.2 and 74.6 eV, respectively. These energies are typically associated with the metallic platinum chemical state.^40,41^

**Figure 10:**
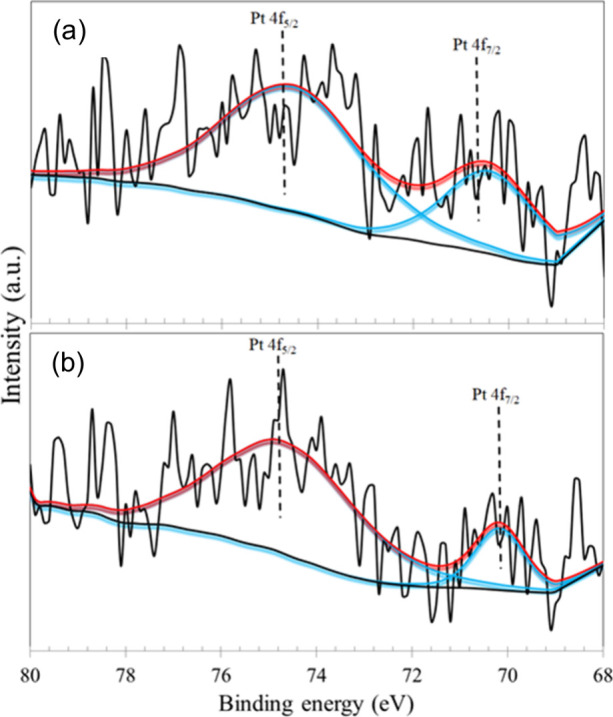
XPS high-resolution spectra of Pt 4f photoelectron lines for the TiO_2_@Pt_0.3%_ samples: (a) before reaction; (b) after reaction.

### Photocatalytic Performance of H_2_ Production

#### Effect of Platinum

As shown in Figure 11, the presence of platinum significantly impacted the photocatalytic production of hydrogen. The isolated application of TiO_2_ in the aqueous glycerol solution resulted in an H_2_ production of 1.4 mmol·h^–1^·m^–2^, while the presence of Pt increased hydrogen production by an average of 227 times. This significant increase in photocatalytic activity is directly related to Pt ability to minimize the recombination of electron/hole pairs through the trapping of electrons by the Schottky barrier mechanism, facilitating their transfer to electron acceptors.^42–44^

**Figure 11:**
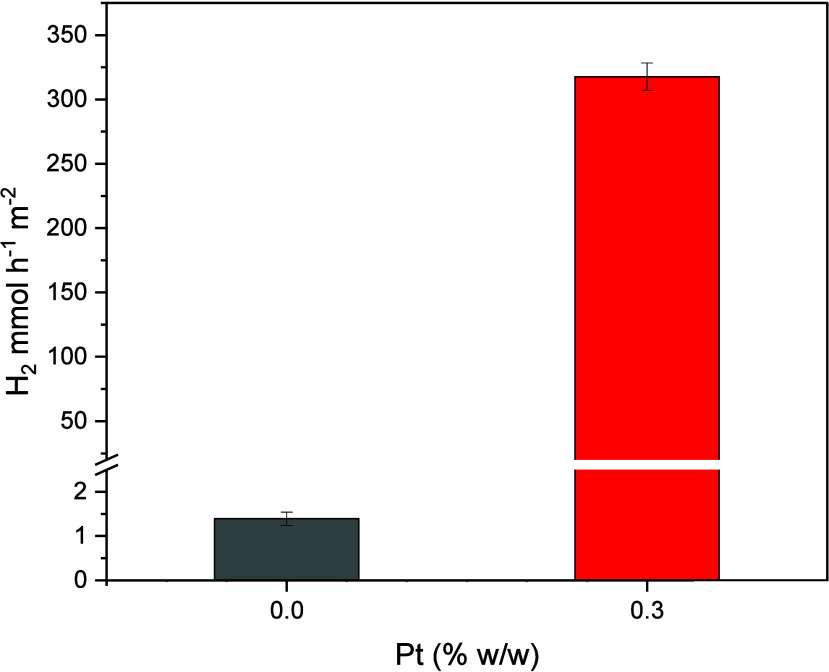
Evaluation of platinum doping on TiO_2_ in the production of H_2_ with photocatalytic plates. *C*_gly_ = 10%, *m*_cat_ = 25 mg (immobilized), *T* = 40 °C, Xe 300 W.

The presence of metallic Pt nanoparticles (zero oxidation state), arising from the reduction of Pt^4+^ on the TiO_2_ surface during the photodeposition process, allows a more efficient separation of charge pairs. These metallic particles act as electron nucleators, preventing their recombination with holes. Pt nanoparticles not only facilitate charge separation but also promote an efficient transfer of electrons to oxygen or other electron acceptors, ensuring a reduction in recombination and, consequently, greater hydrogen production.^45,46^

Several studies have previously reported the use of platinum as a dopant for photocatalytic production of H_2_. Kurenkova et al.^20^ synthesized and tested Pt/TiO_2_ and CuO/TiO_2_ photocatalysts in the photoreforming of glycerol. The reaction was carried out with a catalyst concentration of 0.5 g·L^–1^ and basic glycerol aqueous solution under 6 h of radiation with a UV LED lamp (30 W, λ = 380 nm). The results showed that Pt/TiO_2_ had a hydrogen evolution rate of 1.35 mmol·h^–1^·g^–1^, surpassing CuOx/TiO_2_, which produced 0.55 mmol h^–1^·g^–1^ of H_2_.

Pecoraro et al.^47^ analyzed different polymorphs of TiO_2_, and they concluded that brookite had a greater capacity to adsorb water and that the different distribution of Pt active sites in brookite could positively influence its photoactivity. The catalyst was prepared with 0.5% Pt using the photodeposition method. The process was conducted under UV irradiation for 4 h, and H_2_ production was measured over time. Pt/TiO_2_ anatase had the highest H_2_ production rate, generating 9,300 μmol H_2_·L^–1^ after 4 h of reaction.

Musso et al.^48^ investigated the photocatalytic production of H_2_ from pure and crude glycerol, using TiO_2_ and N-TiO_2_ photocatalysts doped with 1.0% Pt. The results showed that the Pt/N-TiO_2_ photocatalyst was more efficient, producing up to 1,380 μmol H_2_ with UV, 0.92% pure glycerol, and 1,260 μmol H_2_ with crude glycerol.

Fakhrutdinova et al.^49^ analyzed the photocatalytic production of H_2_ from glycerol using dark TiO_2_ modified with Pt, emphasizing the effects of Pt dispersion and the incorporation method on photocatalytic activity. A 20% v/v glycerol solution was used for the photocatalytic tests, with a catalyst concentration of 1.0 g·L^–1^ dispersed in the reaction solution. The results showed that the 0.5Pt­(C)/TiO_2_–Ph system (prepared by photoreduction) presented the highest hydrogen production rate, with 5.26 mmol H_2_·g^–1^ in 3 h of reaction, highlighting the high dispersion of Pt and the presence of Pt^2+^ as key factors in the production of H_2_.

Although the studies present a significant HER,^46,50–53^ the present study stands out due to the amount of hydrogen produced, especially when taking into account the use of a lower amount of platinum (0.3%), as well as by applying the catalyst in immobilized form. This characteristic contributes to reducing catalyst production costs. It makes the process more economical, mainly due to the possibility of reusing the catalyst in successive cycles, emphasizing the importance and practical feasibility of the work. Table 2 shows studies using Pt/TiO_2_ as a catalyst and glycerol as a sacrificial reagent, comparing some parameters of each work.

**Table 2:** Recent Studies on the Photocatalytic Production of H_2_ Using TiO_2_/Pt and Glycerol

photocatalyst	Pt (%)	light source	*C*_cat_ (g·L^–1^)	*C*_gly_ (%)	reaction time (h)	HER (mmol·g^–1^·h^–1^)	reference
TiO_2_/Pt	1.0	30 W LED	0.5	5	10	1.4	^51^
TiO_2_/Pt	1.0	LED light	0.5	20	3	1.3	^52^
TiO_2(P25)_/Pt	1.5	300 W Xe	0.5	7	5	7.0	^16^
TiO_2_/Pt	0.5	Xe lamp	1.0	10	16	18	^46^
TiO_2_/Pt	0.5	LED light	0.5	20	3	5.26	^49^
TiO_2_/Pt	0.45	LED light	1.0	10	6	24.7	^50^
TiO_2_/Pt	0.3	300 W Xe	1.0	10	3	8.6^a^	this work

a Value normalized by immobilized catalyst mass for comparative purposes with other works.

Analysis of the data presented in Figure 11 reveals a significant effect of Pt doping on photocatalytic hydrogen production. Figure 12 shows that, while pure TiO_2_ demonstrates a discrete production of H_2_ with smooth growth, TiO_2_@Pt_0.3%_ shows a considerably higher reaction rate, characterized by exponential growth over 6 h.

**Figure 12:**
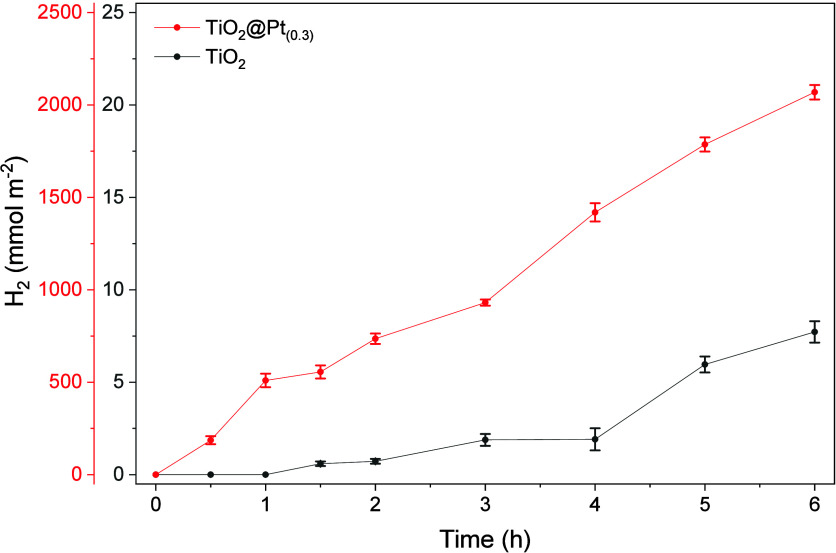
Kinetics of photocatalytic H_2_ production on plates immobilized with TiO_2_ and TiO_2_@Pt_0.3_. *C*_gly_ = 10%, *m*_cat_ = 25 mg (immobilized), *T* = 40 °C, Xe 300 W.

The increase in H_2_ production can be explained by the role of Pt as a cocatalyst, which facilitates the separation of electron/hole pairs, a phenomenon that limits the efficiency of TiO_2_-based photocatalytic systems. Photocatalysis on TiO_2_, although efficient, is still limited in its ability to absorb visible light due to its bandgap (3.2 eV).^54,55^ The kinetic profile of H_2_ production by TiO_2_@Pt_0.3_ suggests that Pt doping not only accelerates the initial rate of H_2_ production but also contributes to the stability of the photocatalytic process over time.^56^ In systems without Pt, the rate of H_2_ evolution was slower, indicating that the process is limited by charge recombination, resulting in a slower reaction rate.

EIS measurements were recorded to evaluate the charge transfer dynamics of the photogenerated species. The Nyquist plots of the catalysts were plotted as shown in Figure 13, in which the diameter of the semicircle is an indication of the material’s resistance to charge transfer, that is, its charge conduction impedance. Thus, a smaller diameter arc is associated with a lower electrical resistance, leading to a more efficient charge separation (e_CB_^–^/h_VB_^+^) with the consequent increase in photocatalytic activity.^57^ As shown in Figure 13, the presence of Pt leads to a reduction in the TiO_2_ semicircle, indicating that the TiO_2_@Pt_0.3_ catalyst had a more effective charge separation mechanism than TiO_2_ alone. This result corroborates the photocatalytic performance presented in Figures 11 and 12.

**Figure 13:**
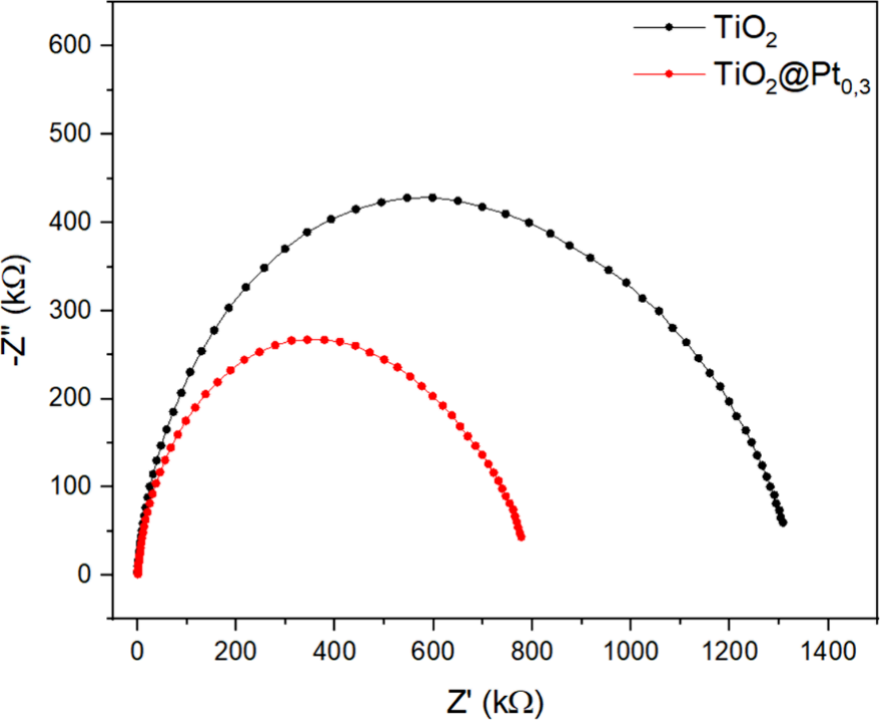
Nyquist plots of TiO_2_ and TiO_2_@P_t0.3_.

#### Influence of Temperature on Photocatalytic Activity

The kinetic behavior of TiO_2_@Pt_0.3_ as a function of temperature is presented in Figure 14. The data indicate that increasing the temperature from 20 to 40 °C resulted in a substantial increase in the rate of H_2_ evolution, with the production of hydrogen at 40 °C being approximately six times greater than the production at 20 °C at the end of the reaction time (3 h).

**Figure 14:**
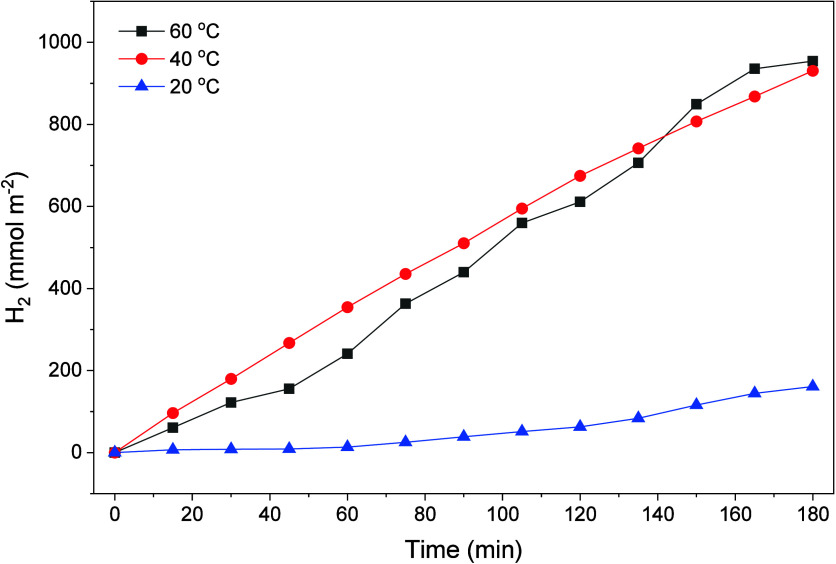
Kinetic profile of photocatalytic H_2_ production as a function of temperature with the TiO_2_@Pt_0.3%_ catalyst. *C*_gly_ = 10%, *m*_cat_ = 25 mg (immobilized), Xe 300 W.

According to Zhong et al.,^58^ the increase in temperature favored the charge transfer mechanism and electrical mobility on the catalyst surface, enabling a more efficient separation of the electron–hole pair. Additionally, the adsorption and desorption processes of H^+^ and other chemical species were thermally variable. Increasing the temperature to 60 °C, however, did not follow the same increase as the previous variation, suggesting that higher temperatures may have limiting effects on the photocatalyst performance. This phenomenon can be explained by the greater electrical resistance of Pt at elevated temperatures, as discussed by Li et al.^59^ The enhanced vibration of metal cations at higher temperatures can result in reduced mobility of free electrons, causing particle agglomeration. Furthermore, increasing temperature can also intensify the photo-oxidation of the substrate, interfering with the adsorption of glycerol on the catalyst surface and leading to a loss in the substrate adsorption step, as reported by Cai et al.^60,61^ These factors contribute to a decrease in the efficiency of the photocatalytic system at 60 °C, limiting the increase in hydrogen production.

#### Byproducts of Glycerol Conversion

Figure 15 shows the concentration of glycerol oxidation byproducts identified under reaction at 40 °C. The main oxidation products identified were dihydroxyacetone and glyceraldehyde, with concentrations of 235 and 249 mg·L^–1^, respectively.

**Figure 15:**
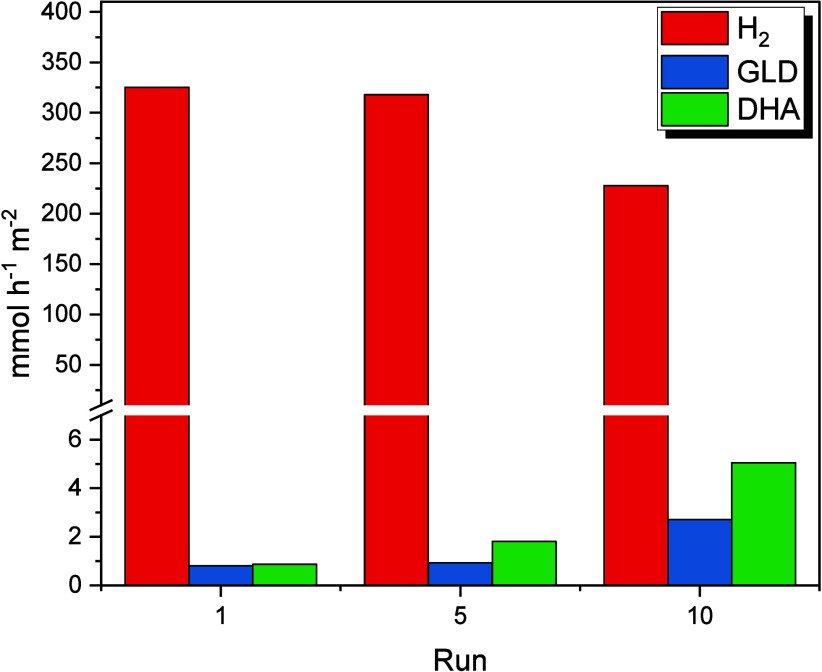
Monitoring of reaction products from glycerol photoreforming under reuse of photocatalytic plates. *C*_gly_ = 10%, *T* = 40 °C, *t* = 3h, *m*_cat_ = 25 mg (immobilized TiO_2_@Pt_0.3_).

The formation of these byproducts can be explained through two distinct reaction mechanisms: (a) a direct pathway, mediated by photogenerated electrons and holes, and (b) an indirect pathway, mediated by reactive oxygen species. In the direct mechanism, photogenerated electrons can react with H^+^ or H_2_O ions to produce radicals, which can react with each other to produce H_2_. On the other hand, the photogenerated vacancies have sufficient potential to promote the oxidation of superficially adsorbed glycerol. In parallel, glycerol can also be oxidized indirectly through the action of reactive oxygen species (ROS) such as hydroxyl radicals (HO^·^), hydrogen peroxide (H_2_O_2_), superoxide (O_2_^·–^), and hydroperoxyl (HOO^·^). ROS formation in an O_2_-free reaction environment can occur through the oxidation of water by photogenerated vacancies, generating hydroxyl radicals, which can recombine to form hydrogen peroxide. Photocatalytic decomposition of the produced H_2_O_2_ can induce the generation of additional ROS, such as hydroperoxide and superoxide radicals, as detailed in eqs 3–9.^62,63^

$$H_{2}O+h^{+}\to {\mathrm{HO}}^{\cdot }+H^{+}$$ 3

$${\mathrm{HO}}^{\cdot }+{\mathrm{HO}}^{\cdot }\to H_{2}O_{2}$$ 4

$$H_{2}O_{2}+e^{-}\to {\mathrm{HO}}^{\cdot }+{\mathrm{HO}}^{-}$$ 5

$$2H_{2}O_{2}\to O_{2}+2H_{2}O$$ 6

$$O_{2}+e^{-}\to O_{2}^{\cdot -}$$ 7

$$O_{2}^{\cdot -}+H^{+}\to {\mathrm{HOO}}^{\cdot }$$ 8

$$H_{2}O_{2}+h^{+}\to {\mathrm{HOO}}^{\cdot }+H^{+}$$ 9

The oxidation of glycerol directly or indirectly leads to the formation of byproducts that may be of interest due to their high added value compared to their original substrate. The mechanism proposed for the photocatalytic valorization of glycerol begins with its dehydrogenation to glyceraldehyde (or dihydroxyacetone).^64^ Future breaks of the C–O, O–H, and C–C bonds can lead to the formation of byproducts in the aqueous phase containing one, two, or three carbons until complete mineralization is achieved with the formation of CO_2_ (Figure 16).

**Figure 16:**
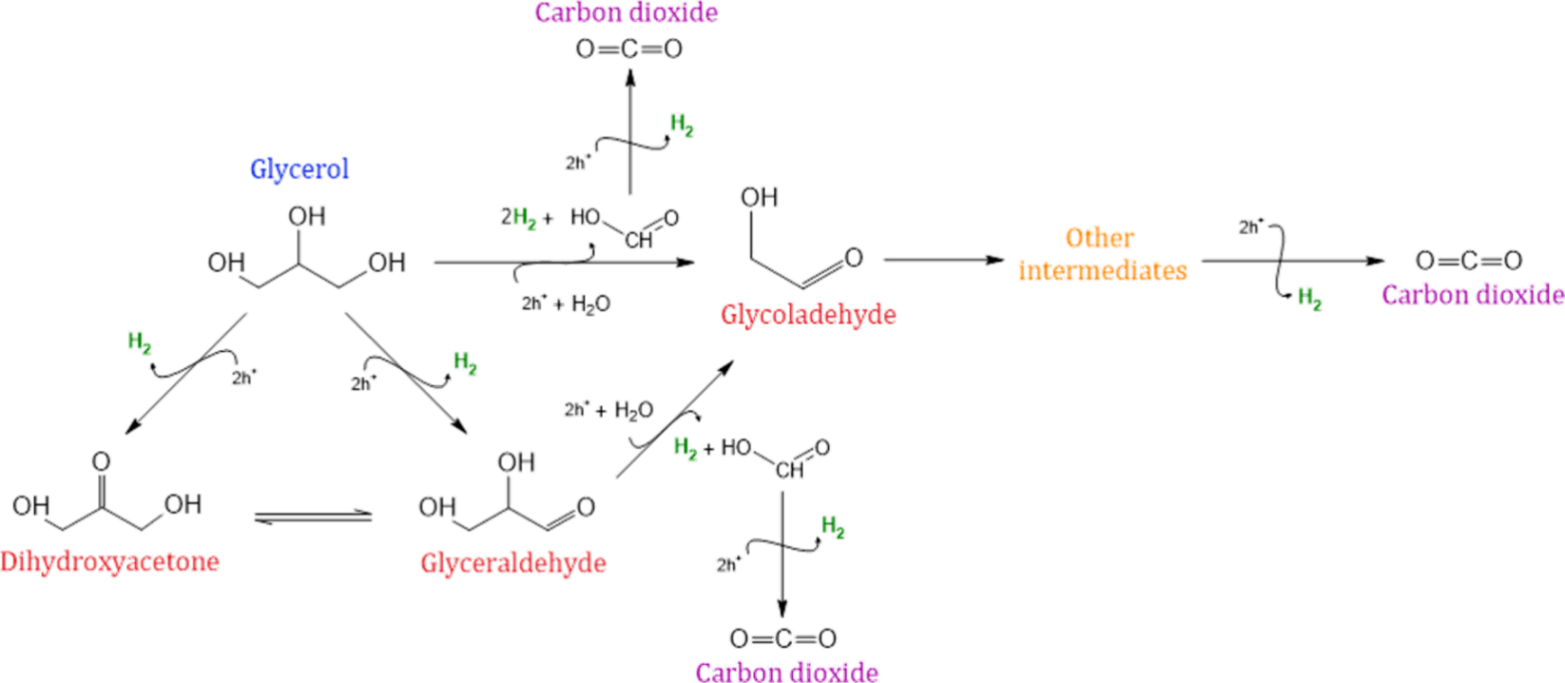
Suggested mechanism of glycerol oxidation.

The indirect mechanism involves the formation of reactive oxygen species, like radicals and peroxides, which leads to the oxidation of glycerol and some high-value-added byproducts, such as glyceraldehyde and dihydroxyacetone. The formation of these compounds suggests that this oxidation route plays a significant role in the photoreforming of glycerol, possibly facilitated by the reactive species generated in the photocatalytic process.

The disparity between the molar yields of H_2_ and the identified primary oxidation products (glyceraldehyde and dihydroxyacetone) indicates that the glycerol oxidation pathway extends beyond these initial steps. According to the stoichiometry of partial oxidation, each mole of glyceraldehyde or dihydroxyacetone formed should be accompanied by one mole of H_2_. The fact that H_2_ production is orders of magnitude higher suggests that most glycerol molecules undergo further oxidative steps toward lower molecular weight carboxylic acids. This hypothesis is corroborated by the pronounced decrease in the pH of the reaction medium (from initial 6.8 to final 3.4 after 3 h), which provides strong experimental evidence for the formation of acidic intermediates, such as formic, acetic, and glycolic acids. Crucially, complete mineralization to CO_2_ appears to be a minor pathway under our reaction conditions. This is supported by our previous work and literature findings, which demonstrate that glycerol photoreforming on similar systems primarily generates H_2_ with only trace amounts of CO_2_, while the liquid phase accumulates a mixture of valuable oxygenated intermediates.^65^ Therefore, the observed mass balance is consistent with a complex glycerol oxidation network dominated by partial oxidation. The process efficiently couples H_2_ evolution with the production of a spectrum of valuable organics in the liquid phase, with minimal carbon loss as CO_2_. This selectivity, evidenced by the significant pH drop, is a key advantage for the valorization perspective of the process.

To evaluate the stability of the catalytic activity and monitor the reaction products, the photocatalytic plates with TiO_2_ were subjected to consecutive application cycles, with no purification procedure between the reuse of the material. According to previously published results,^28^ photocatalytic plates demonstrate stability against H_2_ production. There was a 28% reduction in performance after the 10th reuse cycle, which is an attractive value given the simplicity of operation performed and the absence of a purification procedure. In parallel to the reduction in the H_2_ production rate, the identified byproducts showed an increase in concentration after the continuous reuse of the catalytic plates. This behavior corroborates the observations of Minero et al.,^66^ who also identified these compounds as the leading products in the photoreforming of glycerol.

Direct and indirect oxidation mechanisms play complementary roles in forming these byproducts. The increase in concentrations of glyceraldehyde and dihydroxyacetone in subsequent cycles suggests that the photocatalytic process favors the selective oxidation of primary and secondary alcohols in glycerol. According to the mechanism proposed by Karimi Estahbanati et al.,^64^ the dehydrogenation of glycerol to form glyceraldehyde or dihydroxyacetone is an initial step in the photocatalysis process. The analysis indicates a more significant contribution of the indirect mechanism as the system stabilizes.

## Conclusions

TiO_2_@Pt_0.3%_ immobilized on plates has been demonstrated to be a highly effective photocatalyst for the photoreforming of glycerol, not only due to its ability to produce H_2_ efficiently but also due to high-value-added byproducts formed. The catalyst synthesis and immobilization methods are easily replicable and effectively minimize material loss during reuse cycles, promoting greater operational stability. Immobilization allowed the photocatalytic process to continue without needing constant material recovery, resulting in lower costs and greater long-term efficiency.

The results obtained demonstrate that doping TiO_2_ with Pt (0.3%) using the photodeposition method promoted a significantly higher production of H_2_ and a greater efficiency in charge separation compared to pure TiO_2_, evidenced by the acceleration of the H_2_ evolution rate and the formation of valuable byproducts, such as glyceraldehyde and dihydroxyacetone. The reaction temperature was established as an important condition for increasing the reaction rate. The higher level of photocatalytic activity was, however, reached at 40 °C, with no need to heat the reaction system to increase hydrogen production. The high-added-value byproduct formation reinforces the versatility of the glycerol photoreforming as an important biomass valorization route, using light as the only energy source in the presence of appropriate catalysts.

Using immobilized photocatalysts is advantageous for large-scale photocatalytic processes, ensuring sustainability and industrial applicability. Combining immobilization with control of operating conditions can significantly improve the performance and viability of the photocatalytic process, expanding its application possibilities.

## Supplementary Material





## Notes

Schematic drawing of the photocatalytic reactor (Figure S1) (PDF)
Photocatalytic plate (MP4)
