# Supplementary material for: Photocatalytic Plates for Production of Hydrogen and Value-Added Products via Glycerol Photoreforming
Source: ACS Omega. 2026 Feb 3;11(6):9312–24. doi: 10.1021/acsomega.5c09097 (PMC12917659; doi:10.1021/acsomega.5c09097)
Supplement: Supplementary file 1 [file ao5c09097_si_001.pdf]

Supplementary Material

Photocatalytic Plates for Production of Hydrogen and Value-Added Products Via Glycerol Photoreforming

Mayara Mara Rocha de Oliveira[1], Emanoel Jessé Rodrigues Sousa[1], Luana Sousa Bomfim[1], Mariana Matos Duarte[2], Antônio Jefferson Mangueira Sales[1], Renato Altobelli Antunes[3], Sydney Ferreira Santos[3], Francisco Murilo Tavares de Luna[1], Rinaldo dos Santos Araújo[2], Peter K. J. Robertson[4], Bruno César Barroso Salgado[*2]

[1] Federal University of Ceará. Av. Mister Hull, s/n, Pici, Fortaleza, Ceará, Brazil, 60.455-760

[2] Federal Institute of Education, Science and Technology of Ceará
Av. Parque Central, 1315, Distrito Industrial, Maracanaú, Ceará, Brazil, 61.939-140
* brunocesar@ifce.edu.br

[3] Federal University of ABC. Av. dos Estados, 5001, Bangu, Santo André, São Paulo, Brazil, 09.280-560

[4] Peter K. J. Robertson. Queen's University Belfast, Stranmillis Road, Belfast BT9 5AG, United Kingdom

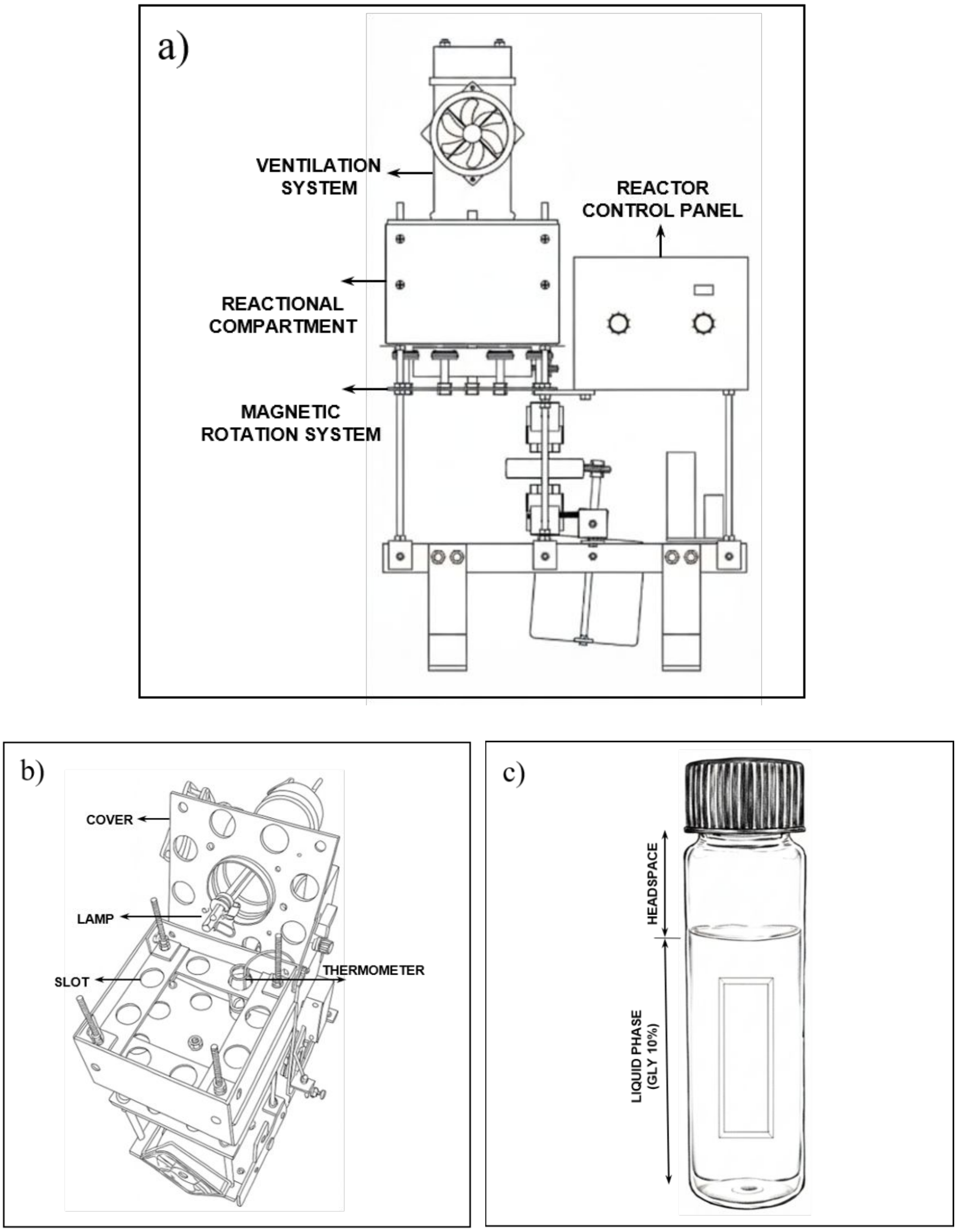

Figure S1. Schematic drawing of the multiple simultaneous reaction system (MSR) (BR 1020210170980): (a) front view, (b) reaction compartment, (c) EPA bottle with silicone septum on the cap, used to accommodate the photocatalytic plate (1.0 x 5.0 cm), filled with 25 mL of 10% glycerol, with a headspace of 15 mL.
